# Individual factors associated with time to non-adherence to ART pick-up within HIV care and treatment services in three health facilities of Zambézia Province, Mozambique

**DOI:** 10.1371/journal.pone.0213804

**Published:** 2019-03-25

**Authors:** Dércio B. C. Filimão, Troy D. Moon, Jorge F. Senise, Ricardo S. Diaz, Mohsin Sidat, Adauto Castelo

**Affiliations:** 1 Provincial Directorate of Health, Zambézia Province, Quelimane, Mozambique; 2 Vanderbilt Institute for Global Health, Vanderbilt University Medical Center, Nashville, Tennessee, United States of America; 3 Department of Medicine, Federal University of São Paulo, São Paulo, Brazil; 4 Retrovirology Laboratory, Federal University of São Paulo, São Paulo, Brazil; 5 Faculty of Medicine, University Eduardo Mondlane, Maputo, Mozambique; University Lyon 1 Faculty of Dental Medicine, FRANCE

## Abstract

**Introduction:**

Mozambique has made significant gains in addressing its HIV epidemic, yet adherence to visit schedules remains a challenge. HIV programmatic gains to date could be impaired if adherence and retention to ART remains low. We investigate individual factors associated with non-adherence to ART pick-up in Mozambique.

**Methods:**

This was a retrospective cohort of patients initiating ART between January 2013 and June 2014. Non-adherence to ART pick-up was defined as a delay in pick-up ≥ 15 days. Descriptive statistics were used to calculate socio-demographic and clinical characteristics. Adherence to ART pick-up was assessed using Kaplan Meier estimates. Cox proportional hazards model was used to determine factors associated with non-adherence.

**Results:**

1,413 participants were included (77% female). Median age was 30.4 years. 19% of patients remained adherent to ART pick-up during the evaluation period, while 81% of patients were non-adherent to ART pick-up. Probability of being non-adherent to ART pick-up by 166 days following initiation was 50%. In univariate analysis, being widowed was associated with higher adherence to ART pick-up than other marital status groups (p = 0.01). After adjusting, being ≥35 years (aHR: 0.843, 95% CI: 0.738–0.964, p = 0.012); receiving efavirenz (aHR: 0.932, 95% CI: 0.875–0.992, p = 0.026); and being urban (aHR: 0.754, 95% CI: 0.661–0.861, p<0.0001) were associated with improved adherence. Non-participation in a Community ART Support Group (CASG) was associated with a 43% increased hazard of non-adherence to ART pick-up (aHR 1.431, 1.192–1.717, p<0.0001)

**Conclusions:**

Interventions should focus on the first 6 months following ARV initiation for improvements. Younger persons and widows are two target groups for better understanding facilitators and barriers to visit schedule adherence. Future strategies should explore the benefits of joining CASGs earlier in one´s treatment course. Finally, greater efforts should be made to accelerate the scale-up of viral load capacity and HIV resistance monitoring.

## Introduction

Mozambique is one of the countries most affected by HIV infection, with a 2015 estimated national HIV prevalence of 13.2% in individuals between 15–49 years of age, translating to approximately 1.5 million adults living with HIV out of a total estimated population of 28 million persons [1–3]. As of December 2017, approximately 1,150,000 persons nationally were receiving antiretroviral therapy (ART). The National HIV/AIDS and Sexually Transmitted Infections (STI) control program of Mozambique (*Programa Nacional de Controle de ITS-HIV/SIDA*) provides ART to all eligible patients free of charge and as of December 2017, ART services were provided in 81% of all health facilities within the National Health System [4].

**Fig 1:**
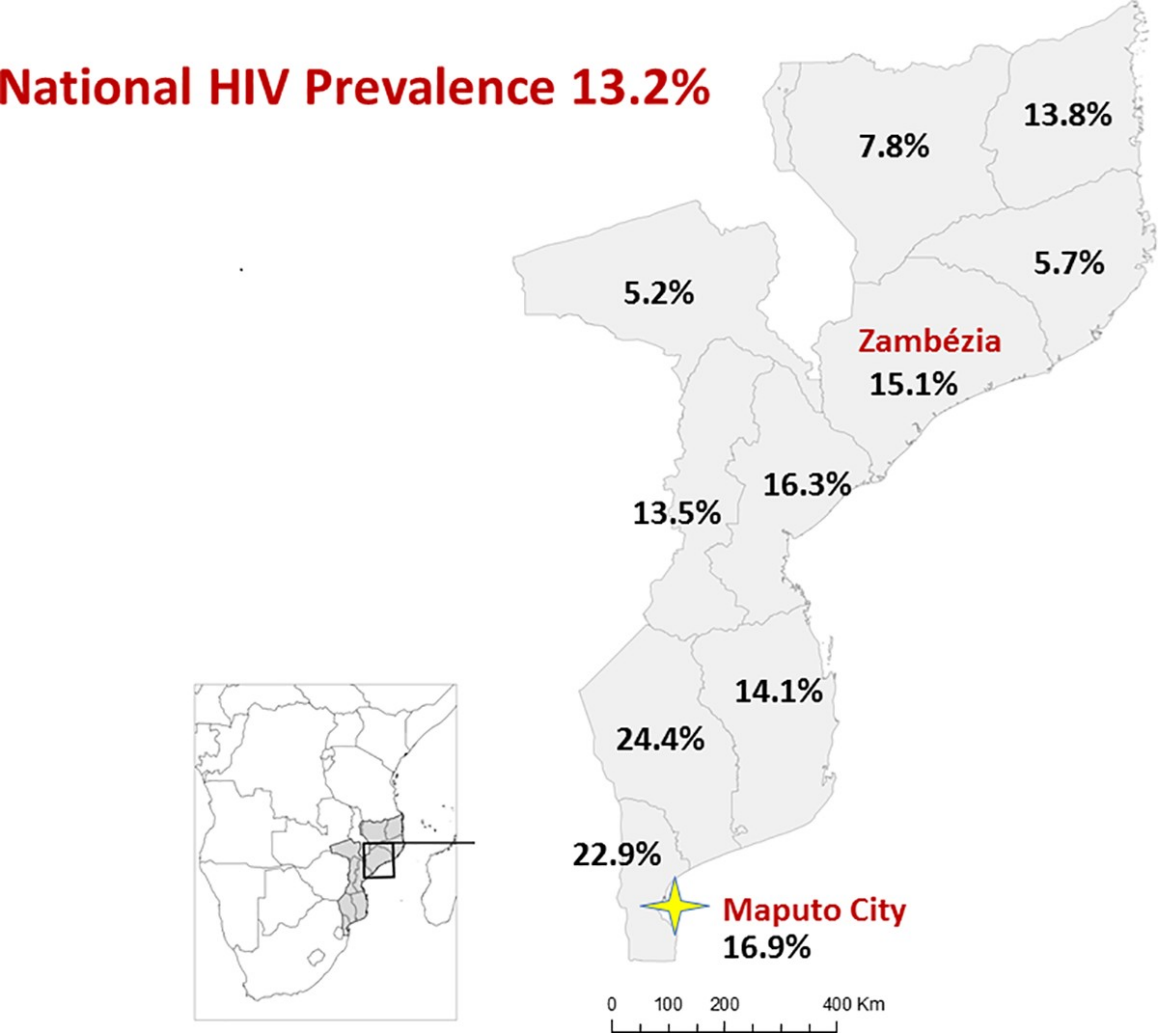
HIV prevalence by province, Mozambique 2015. Data Source: IMASIDA 2015.

Significant efforts have been made by the Mozambican Government to address the HIV epidemic, maintaining a strong commitment to the UNAIDS Fast-Track strategy to end the epidemic by 2030, and in striving to comply with international targets established through programs such as the UNAIDS 90-90-90 initiative by 2020 [2,5–7]. Yet despite this, ART coverage is relatively low with only 54% of all estimated HIV infected persons receiving ART as of the end of 2017 [4]. The magnitude of the HIV epidemic is highlighted in Zambézia Province, Mozambique’s second largest province and home to 4.8 million persons, representing approximately 20% of Mozambique´s total population [8–13]. A mostly rural and underdeveloped province, Zambézia has low literacy rates, poor maternal and child health indices, high rates of tuberculosis and malaria infections, high levels of malnutrition, and comparatively low adult, maternal, and pediatric ART coverage [14–20]. Zambézia Province has the largest number of persons infected with HIV in the country. Provincial HIV prevalence among adults aged 15–49 years, based on the last national prevalence survey in 2015, was estimated at 15.1% overall, 16.8% among women and 12.9% among men (Fig 1) [4].

Low adherence to ART consumption is associated with unfavorable outcomes and can lead to viral resistance to treatment resulting in high costs both to the individual and to national HIV programs [21–23]. Retention in care and treatment is essential to maintaining good adherence, monitoring of treatment outcomes, and providing ongoing psychosocial support and education to the patient [24]. Qualitative and quantitative studies have identified barriers to ART adherence and retention in treatment in Mozambique; including medication side effects, complexity of dosing schedules, low patient education levels, low socioeconomic status, stigma and discrimination, transportation unavailability or cost, simply forgetting, health system quality, and a preference for use of traditional medicines [25–28]. Mozambique´s continued low retention and adherence rates threaten to undo the significant gains made in terms of HIV testing and enrollment in care over the last several years [29]. In Mozambique, it is estimated that upwards of 87% of attrition is due to loss to follow-up (LTFU), with the remaining 13% due to death. Additionally, it is estimated that of patients LTFU across sub-Saharan Africa, between 20–60% are presumed dead, most likely resulting from non-adherence to medication and a rapid progression to disease and death [29–31]. We set out to investigate these issues in order to describe individual factors associated with time to non-adherence to ART pick-up within HIV care and treatment services in three distinct health facilities of Zambézia Province, Mozambique.

## Materials and methods

**Fig 2:**
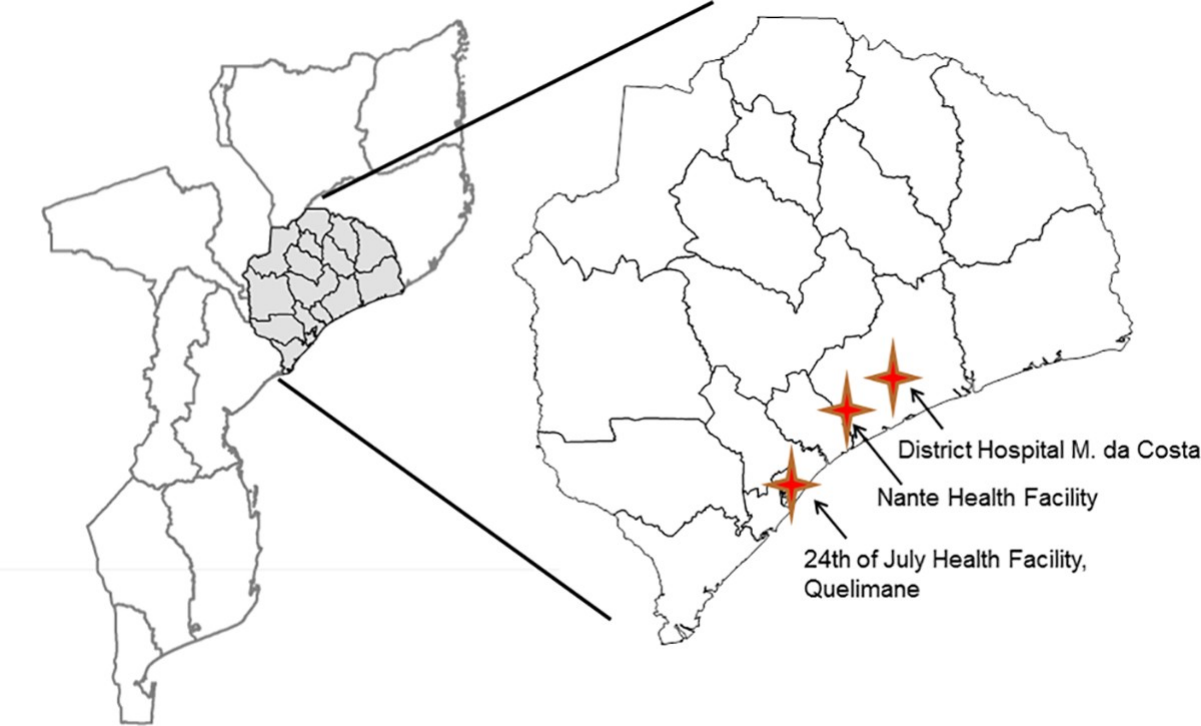
Map of Zambézia Province with location of 24^th^ of July Health Facility, District Hospital of Maganja Da Costa, and Nante Health Facility. *Charlotte Buehler Cherry, November 08, 2017, WGS_1984 Vanderbilt Institute for Global Health.

**Fig 3:**
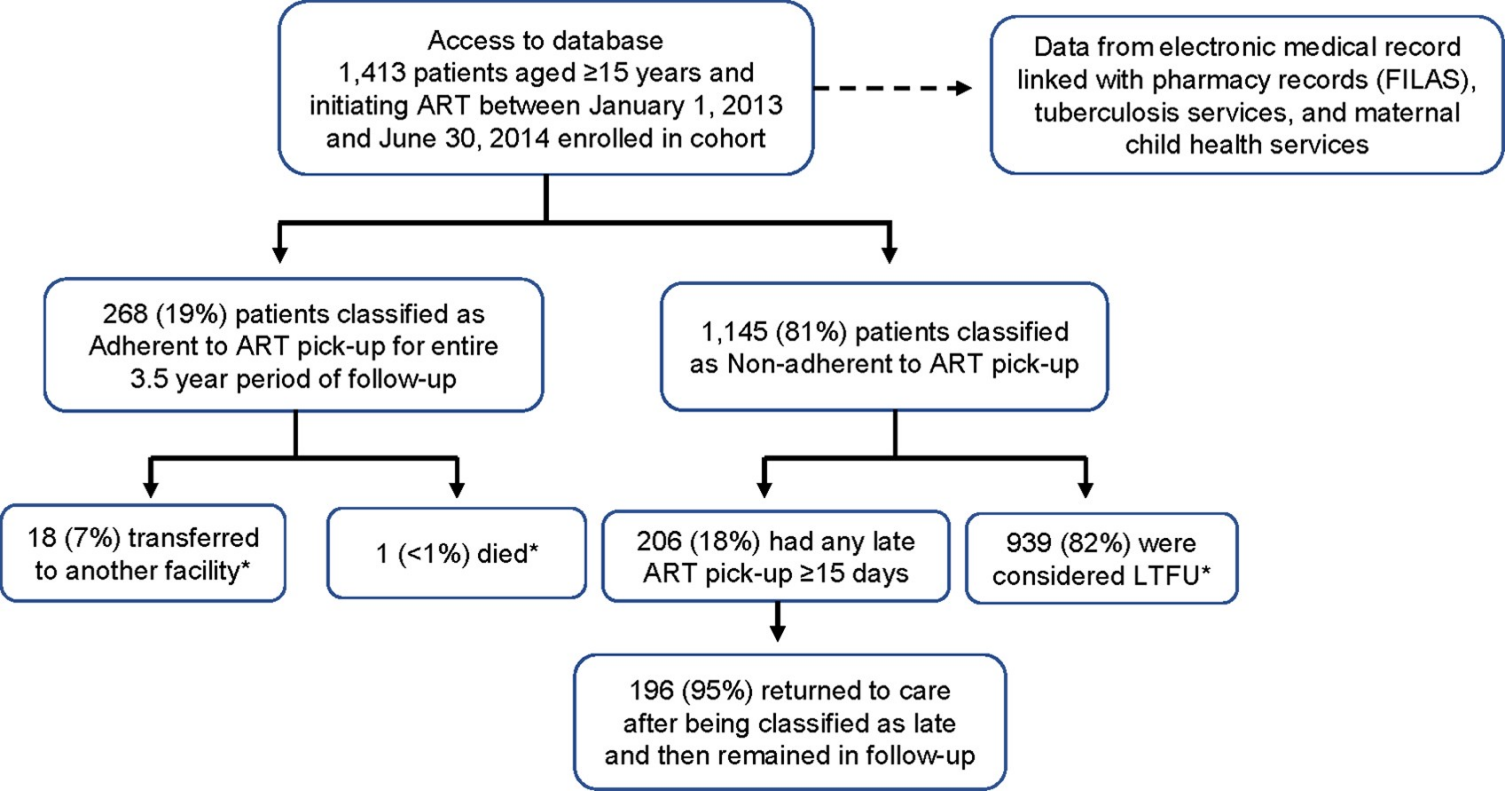
Flow chart of patients enrolled for a minimum of 24 months of follow-up. *Patients LTFU, transferred, or who had died were censored at time of becoming LTFU, transfer, or death.

This retrospective cohort study was conducted in three health facilities of Zambézia Province, Mozambique, representing three different urban-rural zones: *24*^*th*^ *of July*, an urban health facility in the provincial capital city of Quelimane; the *District Hospital of Maganja da Costa*, a rural district capital hospital; and *Nante Health Facility*, an even more rural peripheral health facility in the district of Maganja da Costa (Fig 2). We identified HIV infected individuals at these three sites, aged 15 years or older who started ART between January 1, 2013 and June 30, 2014. For purposes of service delivery, the Ministry of Health (MOH) considers adults to be ≥15 years of age. Each patient was followed from the point of initiation of ART treatment, until June 30, 2016 for a minimum 24-month follow-up (Fig 3).

Data were extracted from patients' paper medical records, filled out by clinicians, counselors and pharmacists, and aggregated into an open source HIV electronic patient tracking system called Open Medical Record System (OpenMRS); including demographic data, clinical information, laboratory results, and pharmacy data. Indicators evaluated included age, sex, education level, profession, marital status, whether urban or rural, health facility, CD4+ T cell count, World Health Organization (WHO) clinical staging, body mass index (BMI), ART regimen, and whether involved or not in a Community Adherence Support Group (CASG).

Data collection comprised two phases. First, authorization was requested to access the database of each of the three health facilities. After obtaining approvals, data was extracted based on eligibility criteria and the variables to be analyzed were exported into Microsoft Excel. Second, data was linked with pharmacy records (called *FILA*, *Formulário de Levantamento dos ARVs*), comparing unique patient identification numbers (NID) of the patient record in OpenMRS and the NID of each FILA. Data were further linked with the NID of patient registers from the tuberculosis services, maternal child health services, and the health facilities death registry. FILAs are a paper-based record that is filled out prospectively at each pharmacy pick-up visit. From each FILA record that the study team could find and then confirm as being a study patient, data were extracted on both scheduled and actual ART pick-up dates for the defined study period. Data systems and patient NIDs are facility specific without ability to track patients across facilities.

Due to limitations in space of each health facility´s pharmacy for archiving records, the health facilities routinely discard FILAs after an unspecified period of time. As a result, it was not possible to manually confirm the ART pick-up dates of all eligible patients identified from the OpenMRS system as planned at study outset. As such, analysis of patient retention for this study was limited to only those patients from which we could link data from the electronic medical record, or other registries, and the FILAs that were located.

Outcomes of interest were calculated by subtracting the actual ART pick-up date from the scheduled ART pick-up date and assumed, based on Mozambican protocols, that the quantity of ART provided was always the same and sufficient for a 30-day supply, regardless if you received your ART at the facility or through a CASG. One exception to this were patients who first initiated an ART regimen during the study period that consisted of AZT+3TC+NVP. These patients only received 15 pills for their first two-week induction. Following this, they received the full 30-day supply as described. While medication stock-outs do continue in Mozambique, it is beyond the capacity and scope of this work to analyze if each patient actually received a 30-day supply of ART or not. We then categorized patients based on the number of uninterrupted days without ART. Patients were categorized as “adherent to ART pick-up” if the number of uninterrupted days was 0–14 days and “non-adherent to ART pick-up” if ≥15 days, our primary outcome of interest. For purpose of analysis, patients who were classified as LTFU, transferred or who had died, were censored at the time of becoming LTFU, transfer or death.

Statistical analysis was performed using IBM SPSS Statistics, version 20 (International Business Machines Corporation Statistical—Package for the Social Sciences). Baseline socio-demographic and clinical characteristics of patients who were adherent and not-adherent to ART pick-up were compared using the chi-square test. Adherence to ART pick-up was assessed using the Kaplan Meier estimate (survival analysis), with graphical visualization of the mean time to first non-adherence to ART pick-up of ≥15 days (survival curve). Patients were followed for a minimum of 24 months but depending on their initiation date it could be longer. Data were censored after 1,278 days (3.5 years) of follow-up. The variables with a significant association with non-adherence to ART pick-up (p< 0.2) in univariate analysis were individually stratified into survival curves (Kaplan Meier) by comparing the two curves using the Breslow test. Continuous variables with significant association with non-adherence to ART pick-up were categorized after analysis using a Receiver Operating Characteristic (ROC) curve to determine the cutoff point that maximizes the division between adherence and non-adherence. Finally, variables significantly associated with non-adherence to ART pick-up in univariable analysis (p<0.2) were analyzed using Cox proportional hazards models. The co-factor Marital Status lost significance in the multivariate analysis and was therefore not included in the final model. The evolution of dropout during the study period was illustrated by Kaplan Meier's estimates in which we used scheduled dates for ART pick-up and added 14 days to those dates, rather than actual dates of ART pick-up. As described above, patients who started an ART regimen during the study period consisting of AZT+3TC+NVP, received a two-week supply of ART for their initial induction period. As such, it is possible for non-adherence to ART pick-up outcomes to occur as early as 30 days post ART initiation.

This study protocol was approved by the Institutional Bioethics Committee for Health of Zambézia Province (*Comité Institucional de Bioética para Saúde- Zambézia*, *CIBS-Z*), as well as the Institutional Review Boards (IRBs) of Federal University of São Paulo (*Universidade Federal de São Paulo*, *UNIFESP*) and Vanderbilt University. Additional approval was obtained from the Provincial Health Directorate of Zambézia Province (*Direção Provincial de Saúde da Zambézia*, *DPS-Z*). All data were fully de-identified prior to accessing them and all three research ethics review committees waived the need for informed consent.

## Results

A total of 1,413 patients aged ≥15 years old were included in analysis for the study period following triangulation between the electronic medical record and the FILA (Table 1). The median age was 30.4 years (IQR: 25.2–36.8) and 77% were female. The majority of patients (65%) lived in rural areas and were either married or involved in a stable relationship (51%). Forty-four percent of patients had achieved only a secondary education or less and 54% were unemployed. The median CD4+ T cell count at treatment initiation was 343 cells/mm^3^ and median BMI was 20.1kg/mm^2^ (IQR: 18.1–22.2). Nearly 67% of patients initiated ART early in their course of disease (WHO Stage I or II). ART fixed dose combinations for 758 (53.6%) patients included Lamivudine (3TC) + Tenofovir (TDF) + Efavirenz (EFV), and for 531 (37.6%) patients included 3TC + Zidovudine (AZT) + Nevirapine (NVP).

**Table 1:** Characteristics of patients ≥15 years old on antiretroviral therapy (ART) enrolled into HIV care between January 01, 2013 and June 30, 2014 at three health facilities of Zambézia Province, Mozambique.

	Total (n = 1,413)
Health Facility n(%)	
District Hospital Maganja da Costa	678 (48%)
Nante Health Facility	247 (17%)
24th of July Health Facility	488 (35%)
Age (Years) median (IQR)	30.4 (25.2–36.8)
Female Sex n(%)	1084 (77%)
Marital Status	
Single	215 (15%)
Married	93 (7%)
Stable Union	619 (44%)
Divorced	1(<1%)
Widowed	73 (5%)
Missing data	412 (29%)
Education	
None	128 (9%)
Primary	335 (24%)
Secondary	153 (11%)
Technical	17 (1%)
University	4 (<1%)
Missing data	776 (55%)
Occupation	
Unemployed	757 (54%)
Employed	192 (13%)
Self employed	198 (14%)
Student	68 (5%)
Retired	2 (<1%)
Missing data	196 (14%)
CD4+ T cell count/mm^3^, median (IQR)	343 (233–538)
Missing data	674 (48%)
BMI (kg/m^2^), median (IQR)	20.1 (18.1–22.2)
Missing data	588 (42%)
WHO Stage	
I	757 (54%)
II	187 (13%)
III	364 (26%)
IV	60 (4%)
Missing data	45 (3%)
ART Regimen	
ZT+3TC+NVP	524 (37%)
TDF+3TC+EFV	749 (52.9%)
Other	140 (10.1%)
Community Adherence Support Group (CASG)	
Yes- participation in	203 (14%)
Adherent to ART pick-up (0–14 days)	268 (19%)
Non-adherent to ART pick-up (≥15 days)	1,145 (81%)

Of the 1,413 patients, only 268 (19%) were classified as adherent to ART pick-up for the entire 3.5-year period of follow-up. Among the 268 adherent patients, 18 (7%) were transferred to another facility and one (<1%) patient had died. The median time of follow-up for these 19 patients was 57 days. The remaining 1,145 (81%) patients were categorized as non-adherent to ART pick-up. Of these, 206 (18%) patients were considered to have had any late ART pick-up of ≥15 days, with 196 (95%) having returned to care following being classified as late and then remained in follow-up. The remaining 939 (82%) patients were considered LTFU. Among patients, a statistically significant higher proportion of non-adherence to ART pick-up came from the rural district of Maganja da Costa as compared to the more urban district of Quelimane (83% versus 77%, p = 0.006). Further still, within Maganja da Costa, when we looked at individual health facilities, a statistically significant higher proportion of patients non-adherent to ART pick-up came from the more peripheral and more rural Nante Health Facility compared to the District Hospital of Maganja da Costa (88% versus 81%, p = 0.001). A statistically significant higher proportion of patients non-adherent to ART pick-up were on an ART regimen comprised of AZT+3TC+NVP as compared to TDF+3TC+EFV (83% versus 79%, p = 0.033). Additionally, a higher proportion of patients non-adherent to ART pick-up did not participate in a CASG (82% versus76%, p = 0.042). Finally, patients non-adherent to ART pick-up were slightly younger with a median age of 30.4 years as compared to 31.1 years for patients adherent to ART pick-up (p = 0.013) (Table 2).

**Table 2:** Patient characteristics by level of adherence to ART pick-up.

	Adherent to ART pick-up* (n = 268)	Non-Adherent to ART pick-up (n = 1,145)	p-value
District			0.006
Maganja da Costa	156 (17%)	769 (83%)	
Quelimane	112 (23%)	376 (77%)	
Health Facility			0.001
District Hospital Maganja da Costa	127 (19%)	551 (81%)	
Nante Health Facility	29 (12%)	218 (88%)	
24th of July Health Facility	112 (23%)	376 (77%)	
Age (Years) median [IQR]	31.1 [25.7–39.4]	30.4 [25.1–36.2]	0.013
Gender			0.822
Male	61 (19%)	268 (81%)	
Female	207 (19%)	877 (81%)	
Marital Status			0.018
Single	49 (23%)	166 (77%)	
Married/stable union	130 (18%)	582 (82%)	
Divorced	0 (0%)	1 (100%)	
Widowed	24 (33%)	49 (67%)	
Education			0.390
None	18 (14%)	110 (86%)	
Primary	62 (19%)	273 (81%)	
Secondary	32 (21%)	121 (79%)	
Technical	1 (6%)	16 (94%)	
University	1 (25%)	3 (75%)	
Occupation			0.552
Unemployed	157 (21%)	600 (79%)	
Employed	33 (17%)	159 (83%)	
Self employed	34 (17%)	164 (83%)	
Student	11 (16%)	57 (84%)	
Retired	0 (0%)	2 (100%)	
CD4+ T cell count/mm^3^, median [IQR]	344 [249–537]	343 [222–539]	0.371
BMI (kg/m^2^), median [IQR]	20.5 [18.1–22.5]	20.1 [18.1–22.1]	0.911
WHO Stage			0.271
I	139 (18%)	618 (82%)	
II	33 (18%)	154 (82%)	
III	82 (23%)	282 (77%)	
IV	9 (15%)	51 (85%)	
ART Regimen			0.033
AZT+3TC+NVP	92 (17%)	439 (83%)	
TDF+3TC+EFV	160 (21%)	598 (79%)	
Community Adherence Support Group (CASG)			0.042
Yes	49 (24%)	154 (76%)	
No	219 (18%)	991 (82%)	

*Adherent to ART pick-up is defined as <15 uninterrupted days without ART

**Fig 4:**
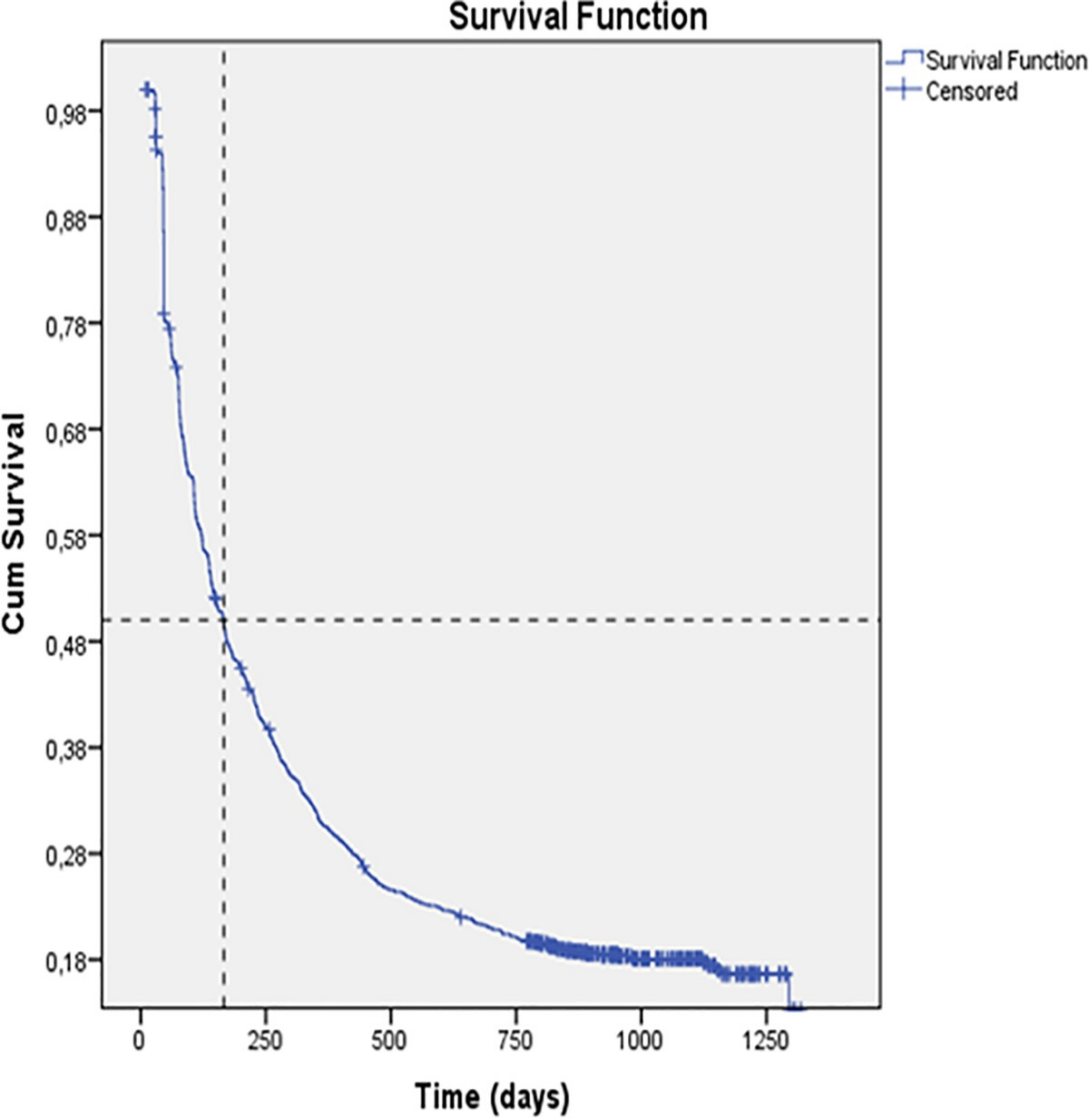
Survival curve for time to first non-adherence to ART pick-up, n = 1,413 patients.

Following initiation of ART, a patient´s risk over time for adherence to ART pick-up dropped rapidly with the probability of being adherent at one month being 96%, yet at three months the probability of being adherent to ART pick-up being only 65%. At 166 days, one year, and two years following treatment initiation, the probability of being adherent to ART pick-up was 50%, 31%, and 20% respectively (Fig 4).

The median time to first non-adherence to ART pick-up of patients younger than 35 years old, was two months less than for older patients (p = 0.008). Patients from Maganja da Costa had a median time for non-adherence to ART pick-up of 4.5 months (140 days) compared to nearly nine months (258 days) for patients from Quelimane (p = 0.001). For patients who participated in a CASG, the median time to non-adherence to ART pick-up was eight months (237 days) compared to only five months (152 days) for those not involved in a CASG (p = 0.001). And finally, the median time for widowers to became non-adherent to ART pick-up was close to one year (346 days) compared to only 5.5 months (169 days) for those either married or in a stable union relationship (p = 0.03) (Table 3).

**Table 3:** Median time in days to first non-adherence to ART pick-up.

n = 1,413	Median days (95% CI)	p-value
Age		0.008
<35	147 (130.4–163.5)	
≥35	215 (177.8–252.2)	
District		0.001
Maganja da Costa	140 (118.2–161.8)	
Quelimane	258 (206.4–309.6)	
ART Regimen		0.057
AZT+3TC+NVP	179 (147.5–210.5)	
TDF+3TC+EFV	168 (143.6–192.4)	
Marital Status		0.033
Widowed	346 (178.4–513.6)	
Married/Stable union	169 (142.6–195.4)	
Community Adherence Support Group (CASG)		0.001
Yes	237 (191.3–282.7)	
No	152 (136.1–167.9)	

In proportional hazards models, factors associated with a decreased hazard of non-adherence to ART pick-up included: being ≥35 years of age (aHR: 0.843, 95% CI: 0.738–0.964, p = 0.012); taking an EFV based ART regimen (aHR: 0.932, 95% CI: 0.875–0.992, p = 0.026); and being treated in the urban area of Quelimane (aHR: 0.754, 95% CI: 0.661–0.861, p<0.0001). Not participating in a CASG was associated with a 43% increased hazard of non-adherence to ART pick-up (aHR 1.431, 1.192–1.717, p<0.0001) (Table 4).

**Table 4:** Proportional hazards models of factors associated with non-adherence to ART pick-up.

	n	aHR (95% CI)	p-value
Age			
<35 years old	885	1.00	0.012
≥35 years old	404	0.843 (0.738–0.964)	
ART Regimen			
Nevirapine-based	531	1.00	0.026
EFV-based	758	0.932 (0.875–0.992)	
District			
Quelimane	247	1.00	<0.001
Maganja da Costa	678	0.754 (0.661–0.861)	
CASG			
Yes	203	1.00	<0.001
No	1210	1.431 (1.192–1.717)	

## Discussion

Retention in HIV care and treatment services and adherence to ART pharmacy pick-up schedules continues to be a significant challenge in Zambézia Province, with only 65% of our study population remaining adherent to ART pick-up after the first three months following treatment initiation. While our study´s poor ART pick-up results are cause for alarm and a need for greater reflection as to what changes can be implemented to help move Zambézia Province´s HIV program towards fully realizing the 90-90-90 initiative, it is important to contextualize these findings based on each study´s inclusion criteria and definition of adherence and retention used. The impact of non-standardized inclusion criteria and definitions of LTFU and retention in HIV programming has previously been shown to impact study conclusions and limit comparability across programs [32]. For example, had the present assessment also been restricted to patients with more than three, six or twelve months of treatment, the observed adherence to ART pick-up rates at twelve months would be 40%, 65% and 67% respectively, rather than the 31% reported here. Policy makers must take this into account when evaluating a program´s reported successes or shortcomings. Our study findings provide additional strong motivation for the establishment of uniform definitions across studies and cohorts to allow for more equitable comparisons.

Determinants of non-adherence to ART pick-up in our study included: being younger (<35 years of age), being treated in a rural area, taking a NVP-based ART regimen, and not participating in a CASG. Furthermore, widowers were more likely to have optimized retention and took almost twice as long to first delayed ART pick-up compared to those who were either married or in a stable union relationship.

Similar to other studies in African countries [33–35], patients <35 years of age in our study were more likely to be non-adherent to ART pick-up overall and became so quicker. Adolescents, a significant portion of our patient cohort, face numerous treatment related challenges that may contribute to the higher rates of non-adherence to ART pick-up observed [36,37]. These include but are not limited to stigma, challenges with disclosure of their HIV status, mental health problems, poverty, and medication-related barriers such as pill burden and side effects [31,38]. In contrast, widowed patients in our study were older and with better adherence to ART pick-up. Overall retention in HIV care and treatment has been shown to be better when the persons HIV status is known and accepted by one´s spouse or family members [34,39]. We can speculate that younger patients, whether they be single or married are more likely to feel the need to hide their HIV status from those close to them or living in the same domicile, and as such could have a harder time maintaining their appointments and subsequently picking up their ARV medications at the pharmacy. Furthermore, it´s likely that widowers who have faced the consequences of HIV infection in their deceased spouses, if that were the cause of the spouse´s death, could have a much different approach to their own health and well-being, resulting in greater retention in care and adherence to scheduled clinic and pharmacy visits, though this has not been previously reported in the literature.

In our study, we utilized routinely collected data and socio-demographic variables for this analysis, which limits our ability to fully ascertain the barriers and facilitators that may influence retention patterns in younger age groups and widows. Future qualitative studies should be implemented that explore these factors more in-depth.

Long distances from the patient’s home to the health facilities and lack of transportation are well described as major barriers to retention in HIV care and treatment in sub-Saharan Africa, especially in rural areas and may explain, at least partially, why adherence to ART pick-up in rural areas is worse in our study [34,40–44]. Much of Zambézia Province´s rural districts, such as Maganja da Costa, have populations dispersed over large geographic regions and consists of subsistence farmers or fisherman, with limited educational opportunities, and who rely on health infrastructure which was heavily damaged or destroyed during Mozambique´s 16-year civil war (1976–1992) [8,15]. In addition, severe health worker shortages, high staff turnover, and fragile health systems further compound the challenges that patients encounter when accessing care in these rural districts [10,45,46]. Since the inception of Mozambique´s HIV program, distance needed to travel to a health facility has been thought to be one of the major barriers to accessing care and patient retention. Taking this into account, in 2012 the MOH initiated an “acceleration plan” to further scale-up HIV programming and address poor ART coverage [45]. This resulted in Zambézia Province increasing the number of health facilities providing ART from 93 at the end of 2013 to 202 at the end of 2016 and thus dramatically reducing the distances patients needed to travel for care [4]. Despite this dramatic advance, retention in care rates for the province have changed little over the same corresponding time period, suggesting that while distance could be a contributor, other factors need to be explored in more detail to determine why it continues to be such a challenge.

Due to the fact that Mozambican protocols recommend monthly ART pick-up at the pharmacy, the strategy of encouraging patients to join or create CASGs reduces the necessity for patients to travel monthly to the health facility pharmacy to pick-up their medication. In consequence, the cost for transportation and the number of times required to go to the health facility is reduced. The impact of being part of a CASG was more visible at the periphery (Nante Health Facility), where the dispersion of the population is over a greater geographic area and public transportation is almost non-existent. It is important to note that based on Mozambican protocols, only patients with at least six months of confirmed retention in care and adherence to medication pick-up schedules should be eligible to join a CASG. If implemented as designed, this criterion would select patients with greater potential for success, since the highest percentage of LTFU occurs in the first six months following ART initiation. Data used for this analysis, however, do not adequately capture the extent to which each CASG adhered to national protocols for membership eligibility and enrollment. Anecdotally it is felt that pressures for rapid national scale-up likely resulted in examples in which persons were enrolled earlier following treatment initiation or who may not have met the strict six-month adherence requirement. Therefore, it is not possible to conclude from our results whether the establishment of CASG at ART initiation would have an impact on Zambézia´s overall poor retention rates during the first six months following treatment initiation.

We found that the time to non-adherence with ART pick-up occurs early after ART initiation, frequently by six months. In Mozambique, patients who are delayed in their medication pick-up or who stopped treatment for a period of time and then eventually restarted ART, received their same prior regimen regardless of the length of time not on ART.

Our data have limitations that affect the completeness of our study. Poor quality of patient registers and poor archiving of pharmacy FILAs limited the number of patients from which we could link data and include in our full analysis. Poor documentation of patient addresses made it not possible to evaluate distance needed to travel to the health facility, as well as to estimate the time and costs associated with accessing care as a potential determinant of non-adherence or poor retention. Additionally, no system currently exists in Mozambique to verify if the patients classified as LTFU are actually seeking care at another health facility. Eligibility requirements for membership within a CASG, if strictly followed, could create inherent biases in selection of persons who have already shown a strong track record of adherence to appointments and retention in care. As such, this must be accounted for when interpreting our results showing a positive association between CASG membership and adherence to medication pick-up. Furthermore, as this was a retrospective and purely quantitative evaluation, it was not possible to explore the impact of stigma on ART pick-up, a factor that is demonstrably relevant in ones accessing of HIV services and treatment. Moreover, this study did not take into account possible interruptions of ART due to adverse effects. Data on side effects, medication availability at the health facility at the scheduled time of pick-up, and mortality which could provide additional valuable insights into understanding the reasons for such low retention in Mozambique were not available. Finally, the severely limited capacity for monitoring viral load and the development of resistance mutations during ART inhibited our ability to document the loss of efficacy of the regimens used as a consequence of non-adherence.

## Conclusion

Mozambique has made significant gains in recent years towards addressing its HIV epidemic and in reaching the first two 90´s of the 90-90-90 initiative. However, continued poor retention in care and poor adherence to medication pick-up threatens its ability to meet the third 90. Interventions aimed to improve ART retention and medication pick-up in Zambézia Province should focus mainly on the first three to six months following ART initiation. Persons <35 years of age with a heightened risk for non-adherence to ART pick-up and widows with better adherence rates are two target groups for future study to better understand factors that are facilitators and barriers to retention. We highlight attainment of CASGs in improving adherence to ART pick-up, but caution is required in over interpreting current implementation strategies due to a potential pre-selection bias for patients eligible for inclusion that have already shown to be retained in care and treatment for six months following treatment initiation. Future strategies should include exploring the benefits of joining CASGs earlier in one´s treatment course. Finally, greater efforts should be made to accelerate the scale-up of viral load capacity and HIV resistance monitoring.
